# Neural Consequences of Increasing Body Weight: Evidence from Somatosensory Evoked Potentials and the Frequency-Specificity of Brain Oscillations

**DOI:** 10.3389/fnhum.2016.00318

**Published:** 2016-06-29

**Authors:** Olivia Lhomond, Normand Teasdale, Martin Simoneau, Laurence Mouchnino

**Affiliations:** ^1^Laboratoire de Neurosciences Cognitives, Centre National de la Recherche Scientifique (CNRS), Aix-Marseille UniversitéMarseille, France; ^2^Département de Kinésiologie, Faculté de Médecine, Université LavalQuébec, QC, Canada; ^3^Centre de Recherche du Centre Hospitalier, Universitaire de QuébecQuébec, QC, Canada

**Keywords:** plantar sole afferents, EEG, standing balance

## Abstract

Previous studies on the control of human balance suggested that increased pressure under the feet, leading to reduced plantar sole mechanoreceptors sensitivity, increases body sway. Although this suggestion is attracting, it is unclear whether increased plantar sole pressure simply reduces the transmission of plantar sole afferent to the cortex or also alters the sensorimotor integrative mechanisms. Here we used electrical stimulation applied under the sole of the foot to probe the sensorimotor mechanisms processing foot mechanoreceptors. Balance control of healthy individuals was assessed either when wearing a loaded vest or in normal-weight condition. In the Loaded condition, we observed decreased cortical activity over the primary somatosensory cortex (SI) for both an early P_50_-N_90_ somatosensory evoked potential (SEP) and for oscillatory brain activity within the gamma band (30–80 Hz). These reductions were interpreted as a disrupted early sensory transmission (i.e., decreased early SEP) leading to a decreased perception of plantar sole sensory information (i.e., decreased gamma band power). These early sensory mechanisms for the Loaded condition were associated with an increase in the late P_170_-N_210_ SEP and oscillatory brain activity within the beta band (19–24 Hz). These neural signatures involved areas which are engaged in sensorimotor integrative processes (secondary somatosensory cortex (SII) and right temporoparietal junction). Altered early and late sensory processes may result from the increase pressure on the mechanoreceptors of the foot sole and not from postural instability *per se*. Indeed, postural instability with normal weight condition did not lead to SEP changes.

## Introduction

It is now well acknowledged that balance is becoming unstable (for instance, increased amplitude and speed of body sway), when plantar sole sensitivity is altered by greater plantar pressures. These modifications of the plantar pressures were observed when normal-weight individuals were loaded with external weights (Vela et al., 1998) as well as in obese individuals (Hills et al., 2001; Gravante et al., 2003; Birtane and Tuna, 2004). Subsequently, studies have shown that balance control is altered by obesity (Hue et al., 2007; Teasdale et al., 2007) and this is not due to muscular weakness, as postural instability in obese sedentary individuals is reported to be similar to highly trained heavy athletes (Handrigan et al., 2012a).

One explanation for the increase oscillations could originate from foot deformation resulting from the extra loading. Indeed it is known that under foot loading the height of the arch of the foot decreases (Bandholm et al., 2008; McPoil et al., 2008) and more than 50% of this change could be accounted by foot arch deformation and less than 50% to skin compression (Wright et al., 2012). Wright et al. (2012) observed that changes in the foot arch height (due to foot loading and unloading) correlates with an increased antero-posterior sway. Mediolateral (ML) sway did not vary with the extra loading. In addition to this mechanical origin of body sway, an alteration of the plantar sensory mechanisms could be involved. Indeed, Handrigan et al. (2012b) in normal weight individuals with an added mass and Wu and Madigan (2014) in obese individuals, reported a lower plantar sole sensitivity to a small and gradual mechanical pressure under participants’ foot sole. While in both of these studies, the gradual change in applied force is known to preferentially activate slowly or ultra-slowly adapting receptors (for review see Delmas et al., 2011), there can be some contribution of the rapidly adapting mechanoreceptors stimulated by an abruptely applied pressure. The slowly adapting receptors concerns solely 30% of the mechanoreceptors in the plantar sole as compared to the high concentration (70%) of the rapidely adapting mechanoreceptors as shown by Inglis and colleagues (Inglis et al., 2002; Kennedy and Inglis, 2002) using microneurography to test the distribution and location of mechanoreceptors in the human plantar sole. It is suggested that functions of these mechanoreceptors as sensors for phasic and tonic stimuli enable sensory neurons to achieve efficient perception of pressure distribution with respect to the base of support.

While the mechanical contributions have been partly verified, it is unknown which stages of sensorimotor processing are modulated by the increase in the plantar pressure. Indeed, the early sensory process has been shown to be related to the incoming sensory inputs and involves the primary somatosensory cortex (i.e., SI, Chapman and Meftah, 2005), whereas late sensory process arising from the secondary somatosensory cortex (i.e., SII) is dependent on the ongoing behavioral demands of the task (Nelson et al., 2004; Saradjian et al., 2013). For exemple, the late integrative sensory processes can be modulated in latency (Altenmüller et al., 1995) or amplitude (Saradjian et al., 2014) when relevant to the task performance. In addition, Chapman and Meftah (2005) have shown, using single unit recordings, that neuronal responses from SI were not changed when attention was directed on the textures scanned under the digit of a macaque monkeys while a large enhancement was observed in SII. The SII region, which comprises the parietal ventral area (PV), has strong connections with the posterior parietal and premotor cortex. Indeed, data from monkeys (Disbrow et al., 2003) and humans (Hinkley et al., 2007) demonstrated that PV receives inputs from and projects to the premotor cortex. This pathway is thought to provide the substrate for sensorimotor integration in tasks requiring continuous processing of feedback such as maintaining upright standing equilibrium with additional weight.

We hypothesized that the early sensory process could have a direct or indirect effect on the late integrative process. We expected both processes to be altered when individuals control their balance while their plantar sole pressure is increased with additional weight (i.e., weight vest, “Experiment 1”). To assess whether the transmission and integration of afferent signals from the plantar sole to the somatosensory cortices were altered, we applied electrical stimulation on the plantar sole to probe the state of plantar sole inputs. It was expected that an increase of the plantar sole pressure would result in a decrease of the early sensory transmission and a depressed late somatosensory integration. However, to disentangle changes in processing due to sensory inputs imposed by the weight vest or due to altered body sway characteristics with external weights, a “sham” experiment (“Experiment 2”) was performed employing a condition of matched sway amplitude in normal weight.

Our approach is built on the current consensus that the sensitivity of the sensory cortex to afferent inputs is shown by an attenuation or a facilitation of the somatosensory evoked potentials (SEPs) recorded by electroencephalographic (EEG) technique (Altenmüller et al., 1995; Duysens et al., 1995). Furthermore, the functional processing of sensory inputs is associated with distinct band-specific neural oscillations within the neocortex. A high power in a given frequency band represents the EEG fingerprint of a synchronized firing pattern of neuron population in this frequency range (Pfurtscheller and Lopes da Silva, 1999). Classically, evoked gamma activity (40-Hz) is strictly phase-locked to the stimulus and is reported as being increased in the visual and somatosensory cortices during visual and proprioceptive stimulation, particularly when these sensory inputs are relevant for the ongoing task (for a review see Tallon-Baudry and Bertrand, 1999). On the other hand, beta oscillations have been associated with the processing of movement–related sensory inputs. Indeed, Tan et al. (2016) have shown that lower power of beta oscillations indicated low confidence in feedforward estimates of the predicted movement outcome and thus the need to rely on the sensory feedback of the actual outcome of the movement. During maintenance of quiet stance, it is conceivable that active stabilization of sway is based on the indirect estimate of a postural state vector obtained from the complex combination of a variety of sway-related sensory signals (Morasso and Schieppati, 1999) with respect to a reference position (Gurfinkel et al., 1995).

We reasoned that analyzing the neural response (i.e., SEP and power of band-specific oscillations) evoked by the cutaneous stimulations would allow for the assessment of the transmission and integrative processes of cutaneous input within the online guidance of body oscillations to maintain equilibrium. In light of this premise, we predicted that the cortical responses to stimulation would be lower when participants are loaded with a weight-vest compared to when they remained unloaded.

## Materials and Methods

### Experiment 1

Sixteen healthy individuals (11 men and 5 women: mean age: 25 ± 3 years; mean height: 174 ± 9 cm; mean weight: 73 ± 13 Kg) participated in this experiment, all giving their informed consent. All procedures were approved by the Ethics Committee at Laval University and all protocols and procedures were also in accordance with the ethical standards set out in the Declaration of Helsinki.

**Figure 1:**
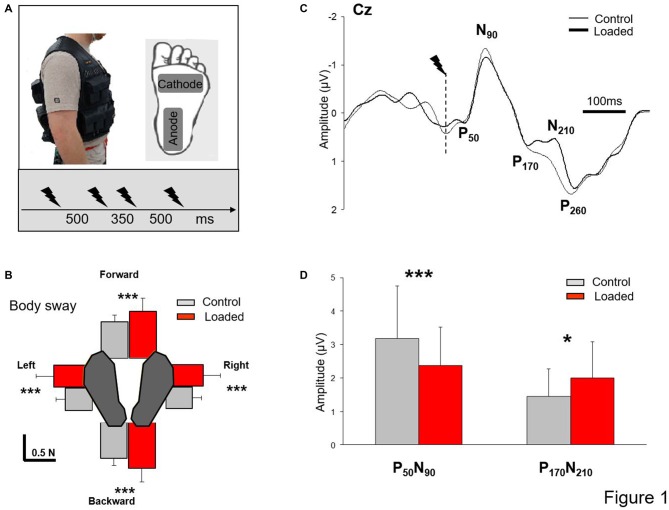
**Somatosensory evoked potential (SEP). (A)** Experimental set up. Insert depicts the workout vest worn by the participants; the added weight was distributed on the front and back of this vest. Position of the stimulation electrodes underneath the left foot and time-intervals between stimulations. **(B)** Mean integral of the horizontal forces in all directions (error bars are standard deviation across participants) ***p < 0.001. **(C)** Grand average SEP for all participants recorded at Cz in both conditions (Control and Loaded). Dashed line indicates the moment of the stimulation.** (D)** Mean for the 60 stimulations of the early and late SEPs amplitudes (error bars are standard deviation across participants) ***p < 0.001 and *p < 0.05. Note that the grand average SEP traces at electrode Cz **(B)** was not representative of the mean value computed for each participant **(C)**.

Before each trial, participants were requested to stand barefoot with their arms alongside their body and to keep their eyes closed. The plantar sole of the left foot was stimulated four times (i.e., St1, St2, St3 and St4) in each trial. To prevent anticipation of the stimulation, we changed the interval between stimuli (Figure 1A); each stimulation was separated by 500 ms except St2 and St3 which were separated by 350 ms. The stimulus was delivered by an isolated bipolar constant current stimulator (DS5 Digitimer, Welwyn Garden City, UK); the cathode was located under the metatarsal region and the anode underneath the heel (5 × 9 cm electrodes, Platinium Foam Electrodes). The stimulation consisted of a single rectangular 10-ms pulse. We used the technique of Mouchnino et al. (2015) who showed that stimulation of the plantar sole skin above the perceptual threshold and below the motor threshold stimulates the plantar sole as a whole rather than targeting a specific portion of the foot. The stimulation intensity was determined as follows: for each participant while in a quiet standing position, we first found the lowest intensity which resulted in a constant perception of the stimulation. This stimulation was determined as the baseline value. The stimulation intensity was set at 25% higher than the baseline value (mean amplitude: 6.9 ± 1 mA). For all participants, this intensity was below the motor threshold.

Participants were asked to stand quietly in two conditions: (i) Loaded, participants were standing while wearing a 19-kg weight vest representing an increased weight of 27 ± 3% (Figure 1A); and (ii) Control, without extra weight. As a result, for the Control condition, participants had a mean body mass index (BMI) of 24.11 ± 2.92 kg/m^2^ (i.e., healthy controls) whereas for the Loaded condition their BMI was 30.25 ± 2.81 kg/m^2^ (i.e., simulating mild obesity for most participants). For both conditions, particular attention was paid to maintain constant the self-selected foot position (i.e., feet shoulder-width apart before each trial) by marking the feet position on the platform. Each participant performed 15 standing trials in each condition (i.e., 60 stimulations). The conditions were presented in block. Half of the participants performed the Loaded condition first while the other half started with the Control condition.

### Experiment 2

Experiment 2 employed a condition matching sway amplitude in the normal weight condition. The procedure and analyses were identical to that in Experiment 1. Six participants (3 men and 3 women: mean age: 28 years; mean height: 174 cm; mean weight: 72 Kg) which were different from the first experiment were asked to stand quietly in three conditions that were counterbalanced across participants: (i) Feet close condition, the feet were close together (i.e., narrowing of the base of support) to increase body sway; (ii) Feet apart condition as in the Control condition of Experiment 1, and (iii) Feet apart condition without electrical stimulation to assess whether electrical stimulation altered body sway. As for Experiment 1, the stimulation intensity was set at 25% higher than the baseline value (mean amplitude: 7.3 ± 1.8 mA). Electrical stimulations (80 stimulations in each condition) were delivered on the plantar sole.

Participants stood, on a 46.4 × 50.8 cm force platform (AMTI OR6–6, Watertown, MA, USA). Ground reaction forces and moments were recorded at a sampling rate of 1000 Hz and used to analyze body sways along the anteroposterior (AP) and ML directions (Figure 1B). Although no specific receptors exists that detect the center of mass, its position and velocity can be indirectly estimated through the horizontal component of the ground reaction forces. Foot skin receptors may have a potential role in assessing these horizontal forces.

Participants were fitted with a Geodesic 64-channel EEG sensor net (GSN64; Electrical Geodesics Inc., Eugene, OR, USA). The EEG was sampled at a rate of 1000 Hz. The electrodes were referenced to the vertex (Cz), and then re-referenced to the net average. We performed data pre-processing with BrainVision Analyzer 2 (Brain Products, Germany). The EEG signals were filtered off-line with 45 Hz (high cut-off) filters (digital filters, 24 dB/octave) and 0.1 Hz (low cut-off) filters (digital filters, 12 dB/octave).

SEPs were obtained by averaging, for each participant and condition, all synchronized epochs relative to the electrical stimulus. The average amplitude of the 60-ms pre-stimulus epoch served as baseline. We measured the SEPs over the Cz electrode as this electrode overlays the sensorimotor cortices and on the homunculus, the feet are located on the inner surface of the longitudinal fissure. The earliest discernible positive (P50) and negative (N90) peaks after each stimulus were identified. These peaks latencies were comparable to latencies evoked by stimulating the sural nerve (Altenmüller et al., 1995; Duysens et al., 1995). The fact that the sural nerve is predominantly a cutaneous nerve (Burke et al., 1981) suggests that P50-N90 originates from cutaneous input. The amplitude of the P50-N90 waveform was measured peak-to-peak (Figure 1C). To estimate the neural sources of the SEPs, we used low-resolution brain electromagnetic tomography (LORETA), implemented in the Brainstorm software (Tadel et al., 2011). The cortical sources were searched at both the early (N1) and late (P170) SEP latencies as measured from the Cz electrode, for each condition and each participant. Distinct brain regions showed enhanced activity at these latencies and were the bases to define the regions of interests (ROIs) used for the source analyses. For each ROI, we computed the mean absolute activity for each participant and condition albeit with some degree of error in estimating the sources of EEG signals due to the absence of MRI data for the head model computation (Michel et al., 2004).

In addition, to asses the different responses of neuronal structures in the brain, the EEG data were transformed into the time-frequency domain using Brainstorm software (Tadel et al., 2011). The time-frequency power of the signals was estimated by means of the Morlet’s wavelet transform (1–100 Hz range of frequencies) of the average response of each participant to clearly identify the stimulus-locked gamma (~30–80 Hz), and beta (19–24 Hz) activity localized to electrode Cz (Neuper and Pfurtscheller, 1996). The signals were then expressed, for each frequency band, relative to a 450 ms window baseline computed in each condition before the stimulus.

### Statistical Analyses

The SEPs (recorded at Cz), ROI and behavioral data were submitted to repeated measures analysis of variance (ANOVA). All dependent variables (EEG and behavioral data) showed normal distributions (i.e., *P*s > 0.05, Kolmogorov-Smirnov test). The level of significance was set at 5% for all analyses.

## Results Experiment 1

### Behavioral Measures of Postural Oscillations

Computing the integral of force over time provides an indication of the momentum of a body and hence of the postural stability. For each trial, baseline values of the horizontal forces (mean for the fore-aft and medio-lateral forces from the trial onset to 3.5 s) were first computed, and the integral of the horizontal forces minus their respective baselines were computed. In this way, the integrals for forward, backward, rightward and leftward forces were calculated during a 2-s period that included all four stimulations (Figure 1B). After normalization to the BMI (including participant’s weight and height), data were submitted to an ANOVA with repeated measures with two conditions (Control and Loaded) and four directions. In the Loaded condition, the integrals of the forces were almost twice higher than in the control condition (*F*_(1,15)_ = 23.7; *p* = 0.0002) irrespective of their direction as confirmed by the absence of an interaction of Load × Direction (*F*_(3,45)_ = 0.33; *p* = 0.79). Also, the integrals of forces were similar in the forward and backward directions (*p* = 0.35) and in the leftward and rightward directions (*p* = 0.98) indicating that the weight distribution did not vary across conditions.

An index of the increased body sway in the Loaded condition has been computed from the mean increase of the four forces in the Loaded condition relative to the Control condition. This index may reasonably be regarded as relating to altered early and late SEPs. The fact that the correlational analyses for the index of increase body sway and the amplitude of the early SEP and late SEP were not significant was not compatible with a putative role of body sway in shaping the SEP waves (Pearson test, *r* = 0.34; *p* = 0.18 and *r* = 0.10; *p* = 0.69 for the early and late SEP, respectively). However, to further disentangle whether changes in processing were due to altered body sway *per se* or to the additional load on the body leading to increase the body sway, a “sham” experiment (“Experiment 2”) was conducted.

### Somatosensory Evoked Potentials

During quiet standing, the foot stimulation evoked typical EEG signals. Figure 1C shows the grand average at electrode Cz for all participants. A small positive component (P_50_) was followed by a prominent negative deflection (N_90_). Thereafter, a notch in the negative-positive deflection occurred at a latency of about 170 ms followed by a late positive component (P_260_). Compared to the Control condition and contrary to our expectation, the notching of the large negative-positive component (N_90_-P_260_) increased and a positive-negative deflection was observed in the Loaded condition (P_170_-N_210_, Figure 1C). This negative deflection was also observed in the Control condition albeit to a lesser extent.

SEP data (amplitude and latencies) were submitted to repeated measures ANOVA with conditions (Control and Loaded) and SEPs components (early P_50_-N_90_ and late P_170_-N_210_ complexes) as the main factor. The results showed a significant interaction between SEPs (early and late SEPs) and conditions (Control and Loaded; *F*_(1,15)_ = 30.50; *p* < 0.05). The analysis of the amplitudes and *post hoc* analyses confirmed that the early SEP (P_50_-N_90_) had a smaller amplitude in the Loaded condition compared to the Control condition (*p* = 0.00046; Figure 1D and Table 1). In contrast, the amplitude of the late SEP (P_170_-N_210_) was greater in the Loaded than for the Control condition (*p* = 0.0063). It was noteworthy that the larger the weight-related changes of the early SEP was (i.e., early SEP decrease), the greater the late SEP increase (Pearson correlation analysis test, *r* = 0.55; *p* = 0.02). No difference was observed for the various latencies (*F*_(1,15)_ = 0.28; *p* = 0.60, for the main condition effect). In addition, we postulated that those results were not due to the variability in the SEP between conditions. Alternatively, they could be due to the fact that the entire individuals-SEPs curve is shifted vertically to the bottom for the early SEPs and to the top for the late SEP in the Loaded condition. This is confirmed by the paired comparison analyses showing a high correlation between conditions (Control and Loaded) either for the early SEP or the Late SEP (*r* = 0.79, *r*^2^ = 0.63; *p* = 0.0002 and *r* = 0.72, *r*^2^ = 0.53; *p* = 0.0013, for the early and late SEPs respectively).

**Table 1:** **Quantifications of the early and late EEG SEPs (mean and standard deviation) in all conditions of the Standing task (NA, non applicable)**.

	Experiment 1		Experiment 2		
	Control	Loaded	Feet apart No stimulation	Feet apart	Feet close
**Early evoked potential**
P_50_ latency	48 ms ± 11	53 ms ± 11	NA	57 ms ± 7	63 ms ± 19
P_50_-N_90_ amplitude	3.2 μV ± 1.6	2.4 μV ± 1.1	NA	2.7 μV ± 1	2.8 μV ± 1.4
**Late evoked potential**
P_170_ latency	168 ms ± 40	174 ms ± 38	NA	188 ms ± 21	183 ms ± 38
P_170_-N_210_ amplitude	1.46 μV ± 0.8	2 μV ± 1.1	NA	1.3 μV ± 0.9	1.28 μV ± 0.8

### Source Localization of the Late SEP

**Figure 2:**
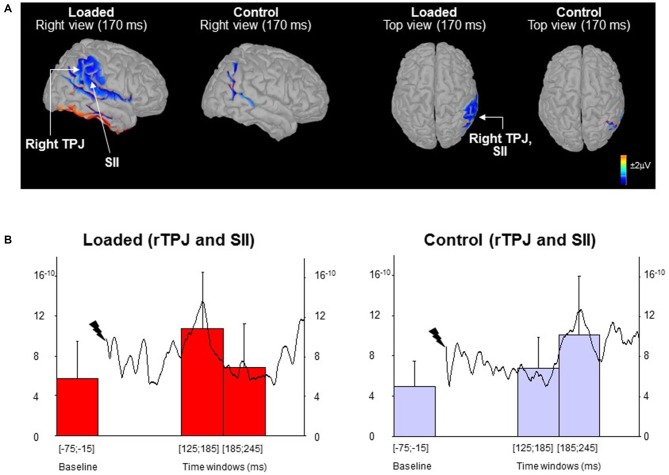
**Source localization. (A)** Topographic maps low-resolution brain electromagnetic tomography (LORETA) computed from all participants’ grand average of the waves (monopolar recordings). The maps are shown for the Loaded and Control conditions at the late P_170_-N_210_ SEP complex latency. **(B)** The curves (right scale) depict the mean activity of the region of interest (ROI) for all participants (*n* = 16) from stimulus to 305 ms post-stimulus. The histograms (left scale) represent the mean ROI activity within each of the three 60 ms-epochs (error bars are standard deviation across participants).

In order to highlight the possible sources of the late sensory process (i.e., P_170_-N_210_), we computed the mean activity of the ROI corresponding to the temporoparietal region during two 60 ms-epochs (i.e., 125–185 ms and 185–245 ms) which encompass the late P_170_-N_210_ component taking into account the between-subject variability in onset latencies. A control epoch of the same duration was computed before the stimulation (hereafter referred to as baseline). Remarkably, brain activity was modulated differently for the temporo-parietal junction (rTPJ) in the right hemisphere (Figure 2A; not observed in the left hemisphere). The latencies of the rTPJ peak activation were submitted to a paired *t*-test (Control vs. Loaded conditions). Figure 2B revealed a peak cortical activity occurring earlier in the Loaded (left panel) compared to the Control (right panel) conditions (*t*_(13)_ = 4.33; *p* < 0.05: 150 ± 31 ms vs. 216 ± 47 ms, respectively). In addition, the mean cortical activation depended on the epochs (*F*_(2,30)_ = 7.90; *p* = 0.0017) and was modulated differently in the Control and Loaded conditions (significant interaction of Condition by Epoch: *F*_(2,30)_ = 6.65; *p* = 0.004). For both conditions, the baseline activities were similar (*p* = 0.59).

### Time-Frequency Analyses of Cortical Oscillations

**Figure 3:**
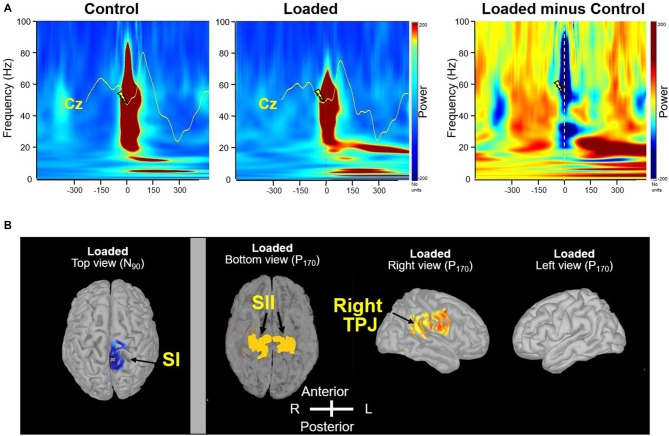
**Frequency-specificity of brain oscillations. (A)** Time-frequency power of the signals by means of a complex Morlet’s wavelet transform applied on the average SEP for each participant, then averaged across participants. Evoked gamma activity is strictly phase-locked to the electrical stimulus. The mean Cz SEP curves were superimposed on time frequency analyses for comparison. Dashed line indicates the moment of the stimulation in the “Loaded minus Control” panel. **(B)** Source localization of beta (20 Hz) frequency band oscillations (right panel). In contrast to primary somatosensory cortex (SI; left panel, top view), secondary somatosensory cortex (SII) showed bilateral activation even with unilateral peripheral activation likely due both to parallel thalamic projections and to serial activation via cortico-cortical and transcallosal fibers (Eickhoff et al., 2007). In the bottom view, the temporal lobes were removed by tranparency artifact for display purposes. The evoked stimulation in the SI showing a lateralized activation at the N90 latency response was included to assess the effectiveness and specificity of applied unilateral left foot stimulation (left panel).

Precise synchronization of the gamma (30–80 Hz) frequency bands is instrumental in subserving a rapid and reliable transmission of information about sensory change and thereby enhancing detection efficiency. As for the early SEP component, the power of the gamma rhythm which was observed in both conditions (Figure 3A), was weaker for the Loaded condition than for the Control condition (*t*_(15)_ = −2.12; *p* < 0.05 at a latency of 88 ms). Afterwards, a clear beta rhythm (17–24 Hz), which is thought to be involved in monitoring the sensorimotor system, was stronger for the Loaded than for the Control condition (Figure 3A; *t*_(15)_ = −2.12; *p* < 0.05 at a latency of 160 ms).

To estimate the brain regions generating the changes in the beta band sensitive to Loading, we identified the sources. The strongest sources accounting for the activity in the beta band were identified in the rTPJ area (Figure 3B, right view) and bilaterally in the parietal operculum (Figure 3B, bottom view).

## Results Experiment 2

Body sway data were submitted to an ANOVA with repeated measures on both factors: three Conditions (Feet close, Feet apart with and without electrical stimulation) and four directions (Left, Right, Forward, and Backward). In the Feet close condition, the integrals of the forces were more than twice higher than in both conditions Feet apart (i.e., with and without stimulation) in the ML directions because the supporting surface was narrowed in that direction (as shown by the Condition × Direction interaction, *F*_(6,30)_ = 5.34; *p* = 0.00074). Besides, the body sway was unaffected by the plantar sole electrical stimulation as no differences were observed between the conditions Feet apart with and without electrical stimulation (*p* = 0.74). These results confirmed that the narrow stance allowed to increase the body sway matching those of the loaded condition of experiment 1. Indeed, the index of increase of body sway did not differed between experiment 1 and experiment 2 (*t*_(20)_ = 1.82; *p* = 0.082).

SEP data were submitted to an ANOVA with repeated measures with two conditions (Feet close and Feet apart with stimulation) and two SEPs components (early P_50_-N_90_ and late P_170_-N_210_ complexes). The amplitude of the early P_50_-N_90_ and late P_170_-N_210_ SEPs did not differ between the Feet close and Feet apart conditions (*F*_(1,5)_ = 0.060; *p* = 0.815). No difference was observed for the various latencies (*F*_(1,5)_ = 0.061; *p* = 0.814). Overall, similarities of the SEP variables between the Feet close and feet apart conditions (“Experiment 2”) suggest that the early and late changes observed in the Loading condition (“Experiment 1”) resulted from somatosensory origin rather than changes in the balance motor commands controlling body sway.

## Discussion

The aim of the study was to determine if the somatosensory transmission from the plantar sole receptors and the sensorimotor integrative processes were altered with an increased in the plantar sole pressure. As expected, compared to the Control condition, the Loaded condition showed a decreased activity over SI for the early process likely indicating a depressed transmission of cutaneous input. Remarkably, this decrease was associated with an increase in the late sensory processes originating from the right temporo-parietal junction (TPJ) and the SII bilaterally.

### Somatosensory Depression During Early Sensory Process

The decrease of the early P_50_-N_90_ SEP in Loaded condition suggests a decrease in the transmission of the afferent cutaneous inflow arising from the periphery to SI. One could argue that the decreased in early SEP reflected changes in sensorimotor processing due to altered body sway. Nonetheless, there is one argument against this suggestion. Result from the second experiment revealed that there was no decreased negativity in the Feet close condition in normal-weight condition despite the fact that body sway amplitudes were similar to the Loaded condition (i.e., “Experiment 1”). As a result, it is suggested that this early brain activity was unaffected by the increased in body sway. In contrast, it would be modulated by changes in plantar sole mechanoreceptors sensitivity. The decrease in sensory transmission observed over the SI is consistent with the presumed role of SI (Chapin and Woodward, 1982; Hämäläinen et al., 1990; Salinas et al., 2000). For example, Salinas et al. (2000) showed that the majority of SI neurons in monkeys were phase-locked with the stimulus input. These neurons encoded the stimulus frequency, suggesting a high relationship of SI activity with the incoming sensory inputs. One possible consequence for the altered early sensory transmission observed in the Loading condition is a decline in perception of tactile stimuli found to be related to the magnitude of early SEP (Duysens et al., 1995). This is in line with Wu and Madigan (2014) study showing a decreased in the perception of the force applied to the plantar surface of the foot among obese individuals. Additional support to the dependence between the decreased early transmission to the cortex and perception comes from the observed decrease in gamma range power (30–80 Hz) in the Loaded condition compared to the Control condition. Indeed, the increased gamma oscillatory process has been correlated with the detection of less salient input irrespective to the sensory afferents involved (e.g., for tactile stimulus in mice, Siegle et al., 2014; and for visual stimulus in monkeys, Womelsdorf et al., 2006). We believe, as suggested by Slobounov et al. (2000), that the synchronous oscillatory activity observed in humans could be a neural marker for detection of unstable balance.

### Enhancement of Later Sensorimotor Integrative Processes

The attenuated transmission of sensory inputs (P_50_-N_90_ SEP) in the Loaded condition was associated with the expression of a late potential (P_170_-N_210_ SEP). The significant correlation between decreased early SEP and increased late SEP suggests that altered sensory transmission enabled the late-stage sensory integration to be enhanced.

The changes in the late SEP component could be primarily due to early sensory afferences disruption imposed by the weighted vest rather than the balance motor commands controlling body sway. As for the early SEP, no modification of the late sensory processes was observed when the state of the plantar sole remained the same but the amplitude of the body sway increased (“Experiment 2”). Furthermore, the altered sensory processes due to the weighting may evoke an increase in body sway (i.e., postural instability). Indeed, Teasdale et al. (2007) have shown that the body sway decreases after weight loss in obese patients (hypocaloric diet or surgery).

The role of the integrative sensorimotor processing in body sway is consistent with the increased activity of the right TPJ and SII which were essentially absent in the Control condition. Indeed, Pérennou et al. (2000) showed that stroke patients with lesions involving the right TPJ showed impaired lateral body stability when sitting on a rocking platform compared with patients with lesions of the same size sparing various areas (e.g., left TPJ, thalamus, frontal or temporal cortex …). Furthermore, this is in line with previous studies (i.e., Blanke et al., 2005; Tsakiris et al., 2008) demonstrating the preponderant role of the right TPJ in the processing of multisensory events that can be (or not) attributed to one’s body motion. In addition, the bilateral activation of S2 could witness the neural coupling of cooperative foot pressure as it was shown in cooperative hand movement (Dietz et al., 2015). For example Schrafl-Altermatt and Dietz (2014) have shown after unilateral forearm nerve stimulation a bilateral activation of S2 solely in cooperative hand movement and not during bilateral hand movements (i.e., non-cooperative). Since S2 is suggested to be involved in the integration of information from the both sides of the body (Lin and Forss, 2002), we hypothesized that S2 is involved in the neural processing of cooperative foot pressures onto the support surface. Although equilibrium maintenance differs basically from hand movements, the neural coupling between lower limbs and feet might be achieved in a similar way.

In the Loaded condition, the larger body sway may have increased the sensorimotor state as shown by the expression of a beta (20-Hz) range activity. Indeed, beta band activity is known to increase when participants are warned of an imminent postural disturbance that they have to counteract (for a review, see Engel and Fries, 2010). This is in line with Simoneau and Teasdale (2015) study showing that the faster body sway observed in obese individuals is related to larger balance motor commands variability. Beta-band activity is central to top-down processing and could interact with bottom up gamma oscillations as suggested by Bressler and Richter (2015) and see Wang (2010), for review. We propose that when there is a low confidence on relying on sensory cues (here low cutaneous transmission) the adaptive behavior is to increase the confidence in feedforward estimate of body sway. Following Tan et al. (2016) conclusion, here, the level of oscillatory synchrony in the beta band serves as an index of confidence in feedforward estimation in sensorimotor control.

Whether body sway could be considered as a disruption in body representation or as a motor control issue remains an interesting question raised by many eminent scientists of the past century, including Gurfinkel et al. (1995) and Massion (1992). For example, Gurfinkel et al. (1995) made a salient observation that although the body sway is controlled to compensate for deviation from a reference position (i.e., motor control issue), the system of postural control needs at least one additional level which elaborates this reference. Indeed, body configuration is stored in memory and its orientation relative to the support should be used as a reference (i.e., body representation issue). In line with their proposal, our current observations may help to unravel the neural processes underlying their hypothesis.

### Limitations of the Study

Some limitation of the present study need to be addressed. First, the additional weight did not represent the same percentage of increase in the mass of each participant. This might introduce a difference in the increase of plantar pressure, and impact the amplitude of the early SEPs in the loaded condition. This, however, is unlikely because we did not find any correlation between the percentage of increased weight and the decrease of the early SEP in the Loaded condition.

More relevantly, future experiments may consider the corticospinal excitability. In our study, the presence of the beta rhythm may be associated with an increased corticospinal excitability. Indeed, Schulz et al. (2014) and Kraus et al. (2016) identified a correlation between corticospinal excitability and beta-band power. Further, the increased body sway observed in our study also suggests an increase of the corticospinal excitability. Indeed, Solopova et al. (2003) reported an increase of the corticospinal excitability when standing on a rocking platform (i.e., leading to greater postural instability). In addition, Taube et al. (2007) showed a decrease of the corticospinal excitability after 4 weeks of balance training.

In conclusion, our results indicate that reduced plantar sole sensitivity depresses the early sensory transmission of afferents from the mechanoreceptors while enhancing the late more integrative processes. These altered neural processes could be at the origin of the increased in body sway amplitude and speed observed in a given population (i.e., obese or weighted individuals such as military troops). This highlights the crucial role of sensorimotor processes and body representation in space in monitoring the balance demands of the task even in a quiet standing position (Lajoie et al., 1993).

## Author Contributions

All authors contributed equally to all aspects of the mansucript. All authors listed, have made substantial, direct and intellectual contribution to the work, and approved it for publication.

## Conflict of Interest Statement

The authors declare that the research was conducted in the absence of any commercial or financial relationships that could be construed as a potential conflict of interest.
